# Glutathione-S-transferase activity in various organs of *Crocodylus siamensis* and its attenuation role in aflatoxin B1-induced cell apoptosis in human hepatocarcinoma cells

**DOI:** 10.14202/vetworld.2022.46-54

**Published:** 2022-01-18

**Authors:** Piriyaporn Thiendedsakul, Pitchaya Santativongchai, Prapassorn Boonsoongnern, Rungrueang Yodsheewan, Phitsanu Tulayakul

**Affiliations:** 1 Department of Animal Health and Biomedical Sciences, Faculty of Veterinary Medicine, Kasetsart University, Bangkok 10900, Thailand; 2 Bio-Veterinary Science (International Program), Faculty of Veterinary Medicine, Kasetsart University, Bangkok 10900, Thailand; 3 Department of Anatomy, Faculty of Veterinary Medicine, Kasetsart University, Bangkok 10900, Thailand; 4 Department of Pathology, Faculty of Veterinary Medicine, Kasetsart University, Bangkok 10900, Thailand; 5 Department of Veterinary Public Health, Faculty of Veterinary Medicine, Kasetsart University, Kamphaeng Saen Campus, Nakhon Pathom 73140, Thailand.

**Keywords:** aflatoxin B1, apoptosis, *Crocodylus siamensis*, glutathione-*S*-transferase, human hepatocarcinoma cells

## Abstract

**Background and Aim:**

The crocodile is a model for studying relevant sources of environmental contamination. They were determined an appropriate biomonitoring species for various toxins. The cytosolic and microsomal fraction of crocodiles plays a role in detoxifying xenobiotics. Cytochrome P450 1A2 (CYP1A2) metabolizes aflatoxin B1 (AFB1) to aflatoxin M1, while glutathione-*S*-transferase (GST) catalyzes carcinogenic agents. This study aimed to investigate the GST activity in various organs of *Crocodylus siamensis*. Further, the fate of microsomal and cytosolic fractions from various crocodile organs against AFB1-induced apoptosis in human hepatocarcinoma (HepG2) cells was investigated.

**Materials and Methods:**

The liver, lungs, intestines, and kidneys tissues from a 3-year-old crocodile (*C. siamensis*) (n=5) were collected. The cytosolic and microsomal fraction of all tissues was extracted, and protein concentrations were measured with a spectrophotometer. Subsequently, a comparison of GST activity from various organs was carried out by spectrophotometry, and the protective effects of CYP450 and GST activity from various crocodile organs were studied. *In vitro* AFB1-induced apoptosis in HepG2 cells was detected by reverse transcription-quantitative polymerase chain reaction. Comparisons between the metabolisms of the detoxification enzyme in organs were tested using the Kruskal–Wallis one-way analysis of variance and Dunn’s multiple comparison tests. All kinetic parameters were analyzed using GraphPad Prism software version 5.01 (GraphPad Software Inc., San Diego, USA).

**Results:**

Total GST activity in the liver was significantly higher than in the kidneys, intestines, and lungs (p<0.05, respectively). The highest GST pi (GSTP) activity was found in the liver, while the highest GST alpha-isoform activity was in the crocodile lung. The kinetics of total GST and GST mu activity in the liver had the highest velocity compared to other organs. In contrast, the kinetics of GSTP enzyme activity was the highest in the intestine. The *in vitro* study of microsome and cytosol extract against apoptosis induced by AFB1 revealed that the level of messenger RNA expression of the Bax and Bad genes of HepG2 cells decreased in the treatment group in a combination of cytosolic and microsomal fractions of the crocodile liver but not for Bcl-2. Interestingly, the downregulated expression of Bax and Bad genes was also found in the microsome and cytosol of crocodile kidneys.

**Conclusion:**

The crocodile liver revealed very effective GST activity and expression of the highest kinetic velocity compared to other organs. The combination of liver microsomal and cytosolic fractions could be used to prevent cell apoptosis induced by AFB1. However, further study of the molecular approaches to enzyme activity and apoptosis prevention mechanisms should be carried out.

## Introduction

Agricultural products are essential components in animal feed production due to the expansion of consumer demand. This increased production can contribute to a higher food contamination risk, including fungal and mycotoxin contamination. Xenobiotics are chemical substances that are not naturally produced but can be found in organisms. These substances can be toxic to the body and reduce the quality of livestock products. For example, contaminant residue in milk, eggs, and meat products can facilitate the production of carcinogens in consumers’ bodies [1]. Fundamentally, drugs or toxins that enter the body undergo biotransformation. If an animal ingests toxic compounds, biotransformation will occur at every metabolic step, including intestinal absorption, entrance to the bloodstream, and excretion from the kidneys. Most biotransformation takes place within the liver. However, some enzymes in this process are located in the intestines, lungs, skin, and kidneys [2].

Aflatoxin B1 (AFB1) is a mycotoxin mainly produced by *Aspergillus flavus* and *Aspergillus parasiticus* [3]. It is a hepatic carcinogen in animals and humans. AFB1 is classified as a Group I carcinogenic agent according to the International Agency for Research on Cancer [4,5]. AFB1 in Phase I is metabolized in the liver by cytochrome P450 (CYP) enzyme, mainly isoenzymes CYP1A2 and CYP3A4 by oxidation to produce many intermediate forms including AFB1-exo-8,9-epoxide (AFBO). AFBO metabolites are produced and bound to DNA molecules. DNA adducts are resistant to DNA repair processes which causes gene mutation; hence, finally development of cancer, especially hepatocellular carcinomas. Phase II reactions are a conjugation of the metabolite AFBO with glutathione (GSH) which are detoxified by glutathione-S-transferase (GST) [6,7]. Although the major target of AFB1 is the liver, it also affects the kidneys and lungs [7-10]. Crocodiles are top predators and long-lived species in their natural environment, containing many contaminants. Therefore, the crocodile is a model for studying relevant sources of environmental contamination [11,12]. Today, freshwater crocodiles (*Crocodylus siamensis*) have become an important and valuable economic resource in Thailand because all organs can be utilized, including the skin, flesh, blood, and fat. Crocodile oil has traditionally been used to treat microbial infections and inflammation [13], the same as crocodile white blood cells and crocodile blood, which have shown antibacterial and anti-inflammatory properties, respectively [14,15]. The crude extracts of crocodile organs also contain active components that affect the viability of prostate cancer (PC3) cells [16]. Moreover, the cytosolic and microsomal fractions of the crocodile liver have played a role in the detoxification of xenobiotics. The microsome fraction (CYP1A2 enzyme activity) can metabolize AFB1 to aflatoxin M1 (AFM1), especially the cytosolic fraction (GST enzyme activity), which produces a protective effect against carcinogenic agents compared to other livestock, poultry, and rodent species [12,17,18].

Therefore, this study compared GST activity in the metabolism of conjugates of xenobiotic substances in various organs of *C. siamensis*, including the liver, lungs, intestines, and kidneys. Microsomal and cytosolic fractions from various crocodile organs against AFB1-induced apoptosis in human hepatocarcinoma (HepG2) cells were also studied *in vitro*.

## Materials and Methods

### Ethical approval

The study was approved by the Institutional Animal Care and Use Committee of Kasetsart University (ID#ACKU61-VET-0202), Bangkok, Thailand.

### Study period and location

The study was conducted from May to December 2020 at the Faculty of Veterinary Medicine, Kasetsart University, Bangkok, Thailand.

### Chemicals

GSH (PHR1359, Sigma-Aldrich, Inc., USA), 1-Chloro-2,4-dinitrobenzene (CDNB) (237329, Sigma-Aldrich, Inc., UK), trans-4-Phenyl-3-buten-2-one (t-PBO) (241091 Sigma-Aldrich, Inc., Germany), ethacrynic acid (EA) (SML1083, Sigma-Aldrich, Inc., USA), cumene hydroperoxide (247502, Sigma-Aldrich, Inc., USA), AFB1 (A6636, Sigma-Aldrich, Inc., Israel), Bio-Rad Protein Assay (Bio-Rad) (Bradford Reagent B6916, Sigma-Aldrich, Inc., USA), bovine serum albumin (12659, EMD Millipore Corp.), HepG2 cells (ATCC^®^ HB 8065™), and fetal bovine serum (FBS) (SV30160.03, HyClone™, Austria) were all obtained from standard commercial-grade companies.

### Animals

Three-year-old crocodiles (*C. siamensis*, n=5) were obtained from a commercial crocodile farm in Nakhon Pathom Province, Thailand. Liver, lung, intestine, and kidney tissues were collected immediately after slaughter with cold chains. Subsequently, the samples were washed, the blood removed by sucrose buffer, and flash frozen in liquid nitrogen. The organs were then stored at −80°C until extractions were performed.

### Cytosolic and microsomal fractions

The cytosolic and microsomal fractions were extracted using a modified method from a previous study [17,19]. Briefly, 4 g of frozen liver, lung, intestine, and kidney tissues were selected and weighed. The tissue was then homogenized in a homogenization buffer (0.25 M sucrose, 0.2 mM DTE, 1 mM EDTA, 10 mM Tris-HCl, pH 7.4). Each sample was centrifuged at 10,000× *g* for 10 min; the pellet was discarded, and the supernatant was centrifuged at 105,000× *g* for 60 min. The supernatant, the cytosolic fraction, was stored at −80°C. Subsequently, the pellet was re-homogenized in a sucrose buffer and centrifuged at 105,000× *g* for 60 min. The supernatant was discarded, and the microsomal fraction pellet was stored at −80°C until use. All of the following steps were carried out at 4°C. The protein concentrations of both fractions were measured by the Bradford method [20] using a protein assay reagent (Bio-Rad Protein Assay, Bradford Reagent catalog number B6916, Sigma-Aldrich, Inc.). Bovine serum albumin was used as the standard protein. The protein concentrations were determined using an iMark microplate reader S/N 11706 (ultraviolet-visible spectrophotometer) measurement at 595 nm.

### GST activity

Detection of GST activity of each substrate was measured using a Spark™ 10M multimode microplate reader 2015 (Tecan Trading AG, Switzerland) and an ultraviolet-visible spectrophotometer at 25°C according to the previous study [21,22] with modifications. Briefly, the cytosolic fractions (1 mg protein) were added to start the reaction with each substrate (CDNB, EA, t-PBO, and calcineurin homologous protein [CHP]). In the GST activity toward CDNB, the reaction mixture contained 1 mM CDNB (substrate for total GSTs), 1 mM GSH, and 0.1 M phosphate buffer pH 6.5, absorbance at 340 nm. The reaction mixture of GST activity toward CHP (substrate for GST alpha-isoform [GSTA]) contained 1.5 mM CHP, 1 mM GSH, and 0.1 M phosphate buffer pH 7, absorbance at 340 nm. In the GST activity toward EA (substrate for GSTA), the reaction mixture contained 0.2 mM EA, 0.3 mM GSH, and 0.1 M phosphate buffer pH 6.5, absorbance at 270 nm. The reaction mixture of GST activity toward t-PBO (substrate for GSTA) contained 0.05 mM t-PBO, 0.25 mM GSH, and 0.1 M phosphate buffer pH 7, absorbance at 290 nm.

### Cell culture and AFB1 concentration

HepG2 cells (ATCC^®^ HB 8065™) were cultured in Dulbecco’s Modified Eagle Medium (DMEM) supplemented with 10% FBS. The incubation conditions included 5% CO_2_ at 37°C and 95% air atmosphere at constant humidity. Cells were subcultured routinely twice a week after trypsinization in a 1:4 split ratio. The final AFB1 concentrations for testing were achieved by adding AFB1 to the culture medium with final acetonitrile (AFB1 solvent) concentration ≤40% (v/v). Briefly, the HepG2 cells were plated in 24-well culture plates with DMEM containing 2% FBS at a density of 6×10^4^ cells/well. After the cells reached 80% confluence, the culture medium was replaced with a fresh medium containing serial dilutions of AFB1 from 20 to 100 ppm (mg/mL). The mycotoxin was exposed for 24 h; neither the medium nor the mycotoxin was replenished during the exposure time. After 24 h of exposure, the percentage of dead cells was counted and calculated for TCID50 determination of the mycotoxin. Appropriate controls containing the same number of solvents were included in the experiment.

### *In vitro* cytotoxicity

Cytotoxic effects were determined in HepG2 cells. Briefly, the HepG2 cells were plated in 24-well culture plates under the same conditions as above. The cells were then cultured in fresh medium containing an appropriate concentration of AFB1 and 0.5 mg protein of liver cytosol, liver microsome, lung cytosol, lung microsome, intestine cytosol, intestine microsome, kidney cytosol, kidney microsome, a mixture of liver cytosol, and microsome, and a mixture of kidney cytosol and the microsome of a crocodile. The conditions were exposed for 4, 6, 12, and 24 h. At the end of the experiments, the reactions were stopped by adding an appropriate volume of buffer RLT of the RNeasy^®^ Mini Kit (Qiagen, Inc., Hilden, Germany).

### Reverse transcription-quantitative polymerase chain reaction (RT-qPCR)

The total RNA in HepG2 cells was extracted using an RNeasy^®^ Mini Kit (Qiagen, Inc., Hilden, Germany). RNA (1 mg) was reverse transcribed into cDNA according to the Luna^®^ Universal One-Step RT-qPCR kit (New England Biolabs Inc., Ipswich, MA, USA). The synthesized cDNA was stored at 80°C. A quantitative reverse transcription-polymerase chain reaction (RT-PCR) determined the messenger RNA (mRNA) levels for Bad and Bax in each sample. RT-qPCR was conducted with an iTaq™ Universal SYBR^®^ Green Supermix (Bio-Rad Laboratories Inc., Hercules, CA, USA) on a CFX96 Touch Deep Well RT-PCR System (Bio-Rad Laboratories Inc., Hercules, CA, USA). The reaction conditions were as follows: Reverse transcription at 55°C for 10 min, initial denaturation at 95°C for 1 min, a total of 40 cycles of denaturation at 95°C for 10 s, and extension at 60°C for 30 s. The designed primers are shown in Table-1. The 2-DDCt method was used to calculate the relative mRNA level of each gene [23].

**Table 1:** Primer sequences for RT-qPCR.

Gene	Sequence
*BAD*	F: 5’- ACGTAACATCTTGTCCTCACAG-3’
	R: 5’- CGATGATGCTTGCCGGAG-3’
*BAX*	F: 5’- GGTGGTTGGGTGAGACTCCT-3’
	R: 5’- GATCTGAAGATGGGGAGAGGG-3’
*BCL-2*	F: 5’- CTTTGAGTTCGGTGGGGTCA-3’
	R: 5’- GGGCCGTACAGTTCCACAAA-3’
*Casp2*	F: 5’- CAGCATGTACTCCCACCGTT-3’
	R: 5’- GCCAGCTGGAAGTGTGTTTG-3’

RT-qPCR= Reverse transcription quantitative polymerase chain reaction; F= Forward; R= Reverse; BAD= BCL-2 associated agonist of cell death; BAX= BCL-2 associated X protein, apoptosis regulator; BCL-2= B cell leukemia/lymphoma 2; Casp2, Caspase 2

### Statistical analysis

Comparisons of GST activities toward the substrates of different organs were performed. The data were presented as mean±standard deviation, and the significance level was set at p<0.05. Comparisons between the metabolisms of the detoxification enzyme of the organs were made using the Kruskal–Wallis one-way analysis of variance plus Dunn’s multiple comparison tests. All kinetic parameters were analyzed using GraphPad Prism software version 5.01.

## Results

### GST activity

**Figure-1:**
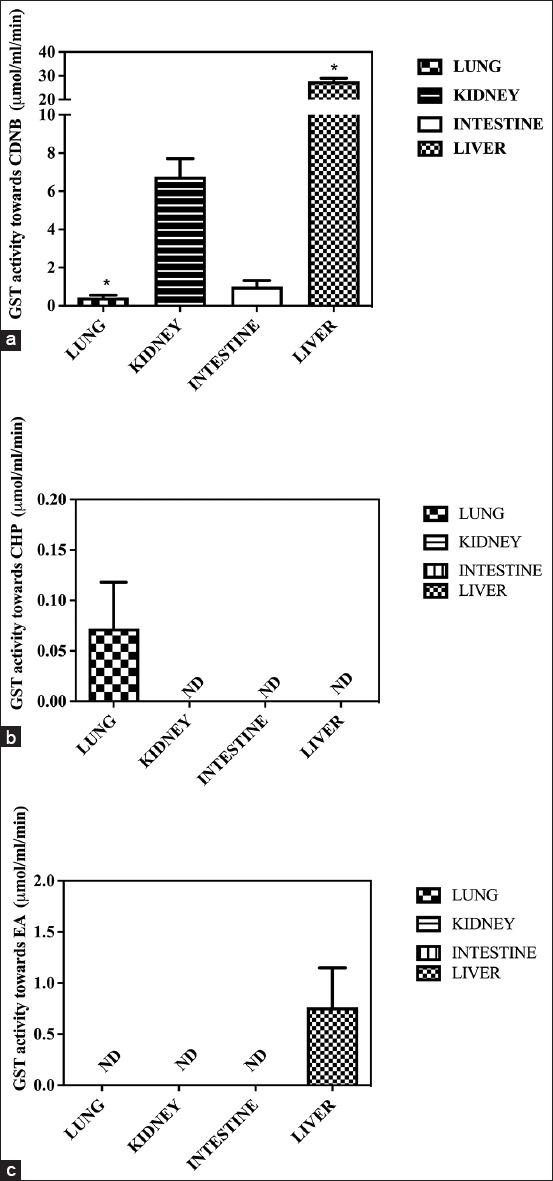
Comparison of glutathione-*S*-transferase (GST) activity toward 1-chloro-2,4-dinitrobenzene (a), calcineurin homologous protein (b), ethacrynic acid (c), and trans-4-Phenyl-3-buten-2-one (d) in different organs. The data are presented as GST activity (mean±standard deviation). The values in brackets represent statistical differences: *Significantly lower (p<0.05).

A spectrophotometer was used to compare GST activities toward CDNB, CHP, EA, and t-PBO in different organs [21,22]. The GST plot against each substrate is shown in Figure-1. The highest GST activity toward CDNB was found in the liver and then in the kidneys, intestines, and lungs, respectively (p<0.05), as shown in Figure-1a. The highest GST activity toward CHP was found in the lungs, as shown in Figure-1b. Moreover, the highest GST activity toward EA was found in the liver (p<0.05), as shown in Figure-1c. However, GST activity toward t-PBO was not detected in all crocodile organs.

**Figure-2:**
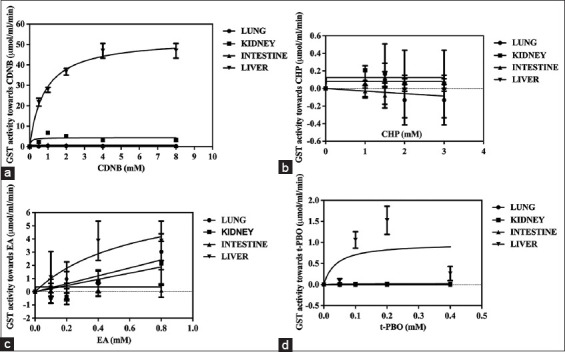
This image demonstrates the Michaelis–Menten plots of cytosolic glutathione-*S*-transferase activity against 1-chloro-2,4-dinitrobenzene (a), calcineurin homologous protein (b), ethacrynic acid (c), and trans-4-Phenyl-3-buten-2-one (d).

The Michaelis–Menten plots of GST activities against the substrates (CDNB, CHP, EA, and t-PBO) in different organs are shown in Figure-2. The Km levels, Vmax, and Vmax/Km ratios were calculated by converting data into Lineweaver–Burk plots. The calculated results are presented in Table-2. The highest kinetic activity (Vmax/Km ratio) of GST activity toward CDNB was revealed in the liver and then the kidneys, lungs, and intestine, respectively (p<0.05), as shown in Table-2. Moreover, the highest Vmax/Km ratio of GST activity toward CHP was found in the kidney, although it was not detectable in the lung. On the other hand, the Vmax/Km ratio of GST activity toward EA in the intestines was higher than in the kidneys and liver. In addition, the highest Vmax/Km ratio of GST activity toward t-PBO was found in the liver (p<0.05), as shown in Table-2.

**Table 2:** Kinetic activity (Vmax/Km ratio) of GST activity toward CDNB, CHP, EA, and t-PBO in different organs.

Species	Organ	GST activity: CDNB			GST activity: CHP			GST activity: EA			GST activity: t-PBO		
			
		Vmax (µmol/min/mg protein)	Km (mM)	Vmax/Km ratio (ml/min/mg protein)	Vmax (µmol/min/mg protein)	Km (mM)	Vmax/Km ratio (ml/min/mg protein)	Vmax (µmol/min/mg protein)	Km (mM)	Vmax/Km ratio (ml/min/mg protein)	Vmax (µmol/min/mg protein)	Km (mM)	Vmax/Km ratio (ml/min/mg protein)
Crocodile	lung	2.72	5.44	0.49	0.03	-1.45	-0.04	0.49	-0.21	-1.84	-0.29	2.52	-0.05
	kidney	5.60	0.54	10.35	-0.05	-0.72	0.09	0.82	-0.10	1.85	0.00	-0.05	-0.08
	intestine	0.67	0.16	0.25	-0.03	-1.14	0.07	-0.17	-0.15	4.27	-0.01	0.06	0.04
	liver	50.77	0.71	80.02	0.09	-1.77	0.06	2.98	0.03	1.17	1.98	0.09	51.04
Chicken	liver	91.74	1.59	57.8	0.12	3.69	0.03	1.21	0.6	2.01	4.61	0.17	27.4
Pig	liver	71.94	1.59	45.25	0.12	3.69	0.03	4.2	0.51	8.22	0.74	0.11	6.64

### Bad and Bax expression in mRNA levels

**Figure-3:**
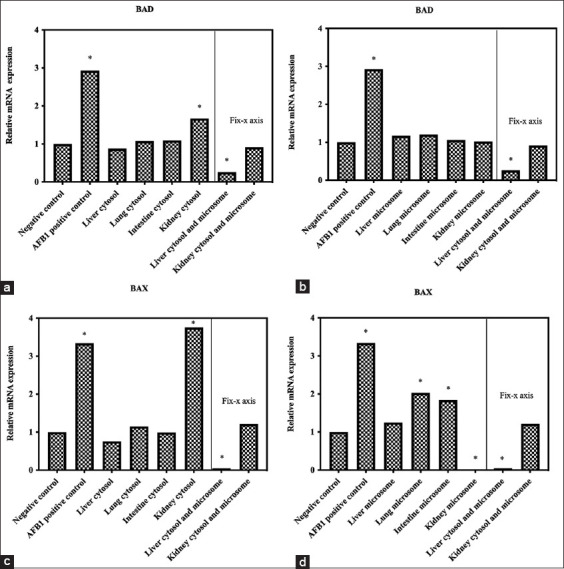
Bad and Bax messenger RNA (mRNA) expressions in human hepatocarcinoma cells incubated with 40 ppm AFB1, cytosolic (a and c), and microsomal (b and d) extracts for 12 h rat. Quantitative reverse transcription polymerase chain reaction analysis was used to examine Bad and Bax mRNA levels. The data are presented as relative mRNA expression. Values in brackets represent statistical differences: *Significantly lower (p<0.01) compared to the negative control.

Bad and Bax expressions at 4, 6, 12, and 24 h of incubations with AFB1 and the cytosolic and microsomal fractions were assessed by quantitative RT-PCR to investigate the detoxification enzyme effect in the crocodile organs on Bad and Bax signaling in AFB1-induced cell apoptosis, as shown in Figure-3. The results showed that a combination of cytosolic and microsomal fractions of the crocodile liver significantly decreased mRNA expression of Bad and Bax in HepG2 cells incubated with 40 ppm AFB1 for 12 h (p<0.01) compared to the negative control group (non-toxic incubation). Moreover, the analysis showed that the microsomal fraction of the crocodile kidney significantly decreased mRNA expression of Bax in cells incubated with 40 ppm AFB1 for 12 h (p<0.05) compared with the negative control group.

## Discussion

The study of GST activity in various organs of freshwater crocodiles (*C. siamensis*) was limited. This pioneering study was a Class II biotransformation called GST activity in the crocodile. Interestingly, total GST activity was highest in the liver, kidneys, intestines, and lungs, respectively. If GST activity is a determinant of species susceptibility, the crocodile is one of the most resistant species to AFB1 toxicity [24]. The liver GST activity in crocodiles was 4.1-fold higher than the kidneys, 64.5-fold than the lungs, and 27.5-fold than the intestine. It was about 2-fold higher than in chickens and pigs compared with other species, as shown in Table-2. These findings are consistent with the previous reports of total GST at the highest levels in the liver, kidneys, and lungs, respectively, in *Rattus norvegicus*. The specific GST pi (GSTP) activity reported in the liver of *R. norvegicus* was 2.4-fold higher and 4.7-fold higher than in the kidneys and lungs. Specific GSTP activity was reported in rats; the kidney was 2.6-fold higher than the liver and 2.4-fold higher than the lungs [25]. The highest GSTP activity was identified in the crocodile liver; it showed undetectable results in other organs. Moreover, Sprague-Dawley rat liver had 5.7-fold higher activity than in the kidneys and 3.4-fold higher than in the duodenum but 3.3-fold lower in the testis. The GSTP activity of the chicken kidneys was 1.2-fold higher than the liver, 1.5-fold higher than the duodenum, and 4.6-fold higher than the testis. A similar trend was found in bobwhite quail [26]. The activity of universal GST was exhibited the highest in the liver > kidneys > lungs and heart in Dhub (*Uromastyx aegyptius*). In contrast, the order was liver > kidneys > adrenals > brain in guinea pigs [27,28]. There is also a report that the highest GST activities (total GST, GSTA, GST mu [GSTM], and GSTP) in Japanese quail were in the kidney compared with the liver, brain, and lung [29]. In addition, the GST activity in fish liver was higher than in the blood [30]. The activity of GST in the liver of cattle, horses, pigs, rabbits, and sheep was higher than the mucosa of the cecum [31]. Even though the highest levels of cytosolic GSTs in humans were in the kidneys rather than the liver, adrenal glands, and blood, conversely, GST activity was reported to be highest in the liver of rats and crocodiles [32,33], which is similar to our findings in crocodiles. These results suggest that liver tissue has the highest antioxidant enzyme activity to counteract oxidative damage [28]. The high metabolic rate of the liver for universal GST plays a key role in the processes of xenobiotic detoxification and enzyme composition specificity. The liver of vertebrates exhibits high metabolism and oxygen consumption, and it is the main organ for xenobiotic detoxification [30]. Our findings revealed that GSTA and GSTP activity was mainly in the lungs and liver but undetectable in other organs. GSTP is mainly expressed in the liver, lung, placenta, breast, and urinary bladder [34,35]. It plays a role in detoxifying and eliminating toxins since it is expressed predominantly in normal epithelial cells of the urinary, digestive, and respiratory tracts [36]. While GSTA is found primarily in the liver, it is also present in the testis, kidneys, and adrenal glands [37]. It produces steroid isomerase activity in rat ovaries and testis [38]. Moreover, it is expressed in steroidogenic tissues [39]. Unfortunately, GSTM activity was not detected in all crocodile organs. Nevertheless, it is found in relatively low amounts in the liver, lungs, brain, heart, spleen, and testis [35,40]. High GSTM expression is an important factor in preventing chemical mutagens and carcinogens [41]. However, the role of specific GSTA isotypes and their mechanism in freshwater crocodiles should be studied further.

The kinetic velocity (Vmax/Km ratio) of GST activity in various organs emphasized that the liver and kidneys were very active in detoxifying xenobiotic exposure. The highest velocity of GST activity toward CDNB in crocodiles was in the liver and then the kidneys, lungs, and intestines, respectively. The highest velocity of GSTM activity toward t-PBO was recognized in the liver. In contrast, the highest velocity of GSTA toward CHP and GSTP toward EA was found in the kidneys and intestines, respectively. The Vmax/Km ratio of GSTP activity toward EA in the intestines was higher than the kidneys and liver, respectively. In some reports, GSTA was involved in Phase II metabolism and was a better marker of hepatocellular injury than renal injury.

In contrast, serum GSTA is a good liver injury recovery marker [42,43]. Moreover, it has been reported that human tumors and human tumor cell lines express a significant amount of GSTP, whereas GSTP overexpression has been found in anticancer drug resistance. Nevertheless, the mechanism responsible for GST overexpression showed marked inter-individual differences in GSTA, GSTM, and GSTT expression [44].

The effect of the detoxification enzymes in various crocodile organs was assessed by studying Bad and Bax signaling on AFB1-induced cell apoptosis. A combination of cytosolic and microsomal fractions of the crocodile liver significantly decreased mRNA expression of Bad and Bax in HepG2 cells, while the expression of Bcl-2 revealed no significant differences. It was recognized that low Bcl-2 expression, attributable to its naturally low expression in the crocodile liver. In contrast to the report by Li *et al*. [45] that mRNA of Baxa was mainly expressed in the liver and ovary of yellow catfish (*Pelteobagrus fulvidraco*), the Baxa was found to be higher in the liver and muscle than in the brain and gills of zebrafish (*Danio rerio*) [46,47]. In aquatic species like fish, many reports have mentioned the high mRNA expression of Baxa and Baxb in the ovary but lower expression in the liver and brain [48]. In yellow catfish, Bcl2 mRNA expression was highest in the brain and mesenteric fat, spleen, kidneys, gill, muscle, heart, liver, and lowest in the ovary and intestines [45]. Meanwhile, Bcl2 mRNA was predominantly expressed in the spleen, kidney, liver, heart, gill, and brain but lowest in the intestine in striped snakehead (*Channa striatus*) [49].

An AFB1 diet induced the decrease of T-cell subsets, morphological changes, and excessive apoptosis of the thymus. The expression of Bax was increased, and the expression of Bcl-2 was decreased in the thymus in broiler chickens [50,51]. Moreover, AFB1-intoxicated chickens showed upregulation of the death receptors FAS, TNFR1, and associated genes and downregulation of inhibitory apoptotic proteins XIAP and Bcl-2 [52]. Furthermore, the toxic effects of AFB1 and AFM1 on kidney tissue in mice treated with aflatoxins showed that proline dehydrogenase and pro-apoptotic factors such as Bax and caspase-3 were upregulated. At the same time, the inhibitor of apoptosis Bcl-2 was downregulated [9]. Interestingly, mRNA expression of p53, caspase-3, Bax, caspase-9, Bcl-2, and cytochrome-C levels in broiler chicken was upregulated in an AFB1-fed group relative to the control group.

Meanwhile, the anti-apoptotic protein Bcl-2 mRNA expression level was markedly downregulated in AFB1-induced apoptosis compared with the control group. However, the study of curcumin supplementation in the broiler study diet showed that AFB1 decreased Bcl-2 mRNA expression level in a dose-dependent manner. Thus, curcumin supplementation prevented AFB1-induced apoptosis in the broiler liver by modulating mRNA expression of apoptotic-related genes [53]. Our study suggested that combining crocodile liver microsomal and cytosolic fractions induced Bad and Bax mRNA expression downregulation. Therefore, reducing cell apoptosis compared with the AFB1 treatment group restores the hepatocyte to normal activity rather than other organs through this pathway.

## Conclusion

The total GST activity was presented in crocodiles, mainly expressed in liver tissue than other organs. Likewise, the kinetic of total GST enzyme activity in the crocodile liver was very active with the highest kinetic velocity compared with other organs. GSTP activity was highly expressed in the liver, while the highest GSTA activity was in the lungs. In contrast, GSTM activity was not detectable in any crocodile organ. Nevertheless, the kinetic velocity of GSTA activity was at a very low level in all organs. However, the kinetic velocity of GSTM activity was high in the liver, while the kinetic of GSTP enzyme activity was highest in the intestines.

Furthermore, the mRNA expression level of the Bax and Bad genes of HepG2 cells decreased with treatment with a combination of microsomal and cytosolic fractions in the crocodile liver. However, they were not effective for microsome or cytosol alone, except for recognizing the downregulation of Bad and Bax gene expression induction in microsomal or cytosolic fractions in the kidneys. Thus, the crocodile liver revealed very effective GST activity and the expression of the best kinetic velocity compared to other organs. The combination of liver microsomal and cytosolic fractions could be used to prevent cell apoptosis induced by AFB1. However, studies concerning the molecular approaches to enzyme activity and apoptosis prevention mechanism should be scrutinized further.

## Authors’ Contributions

PT and PT: Designed the research methodology and supervised the study. PT and PB: Processed and evaluated protein analysis. PT: Processed the measurement of the GST activities in the metabolism of the conjugates by xenobiotic substances among various organs of *C. siamensis*. PS and RY: Processed the measurement of the microsomal and cytosolic fractions from various crocodile organs against AFB1 induced apoptosis in HepG2 cell *in vitro*. All authors read and approved the final manuscript.
